# The pathophysiological role of angiotensin receptor-binding protein in hypertension and kidney diseases: Oshima Award Address 2019

**DOI:** 10.1007/s10157-020-01861-4

**Published:** 2020-02-29

**Authors:** Hiromichi Wakui

**Affiliations:** Department of Medical Science and Cardiorenal Medicine, Yokohama City University Graduate School of Medicine, 3-9 Fukuura, Kanazawa-ku, Yokohama, 236-0004 Japan

**Keywords:** Renin–angiotensin system, Hypertension, Receptor, Kidney diseases

## Abstract

Excessive activation of the tissue renin–angiotensin system through angiotensin II (Ang II) type 1 receptor (AT1R) plays a pivotal role in the pathogenesis of hypertension and related organ injury. AT1R-associated protein (ATRAP/Agtrap) was identified as a molecule specifically interacting with the carboxyl- terminal domain of AT1R. The results of in vitro studies showed that ATRAP suppresses Ang II-mediated pathological responses in cardiovascular cells by promoting AT1R internalization. With respect to the tissue distribution and regulation of ATRAP expression in vivo, ATRAP is broadly expressed in many tissues as is AT1R including kidney. The results of in vivo study employing genetic engineered mice with modified ATRAP expression showed that ATRAP inhibits cardiovascular injuries provoked by Ang II-induced hypertension, along with preserving physiological AT1R signaling. In addition, we have shown that ATRAP functions as an endogenous modulator so as to prevent hypertension in response to pathological stimuli, by regulating renal sodium handling. Furthermore, ATRAP may have an AT1R-independent function of renal proximal tubule to protect aging and fibrosis. These results suggest the clinical potential benefit of an ATRAP activation strategy in the treatment of hypertension and cardiorenal and vascular diseases.

## Introduction

**Fig. 1:**
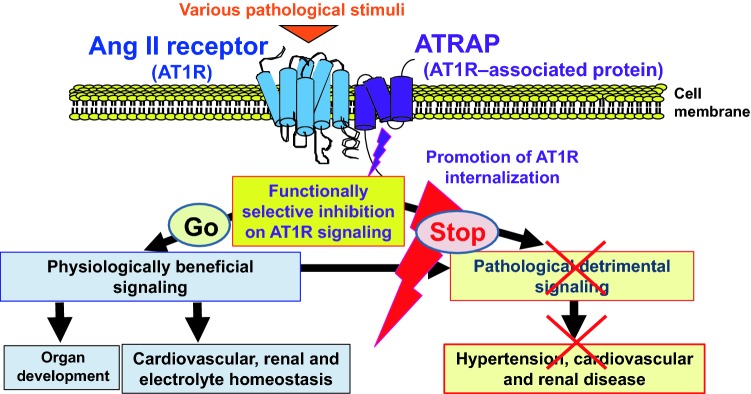
ATRAP inhibits pathological hyper-activation of angiotensin type 1 receptor signaling, but preserves physiological angiotensin type 1 receptor signaling

Accumulating evidence shows that the hyper-activation of the tissue renin–angiotensin system (RAS) through angiotensin II type 1 receptor (AT1R) plays a pivotal role in the pathogenesis of hypertension and associated end-organ injury. On the other hand, physiological AT1R signaling is essential for maintaining organ homeostasis. For example, complete deficiency of angiotensin peptides reportedly provokes severe hypotension and abnormalities in renal structure and function at birth [1]. Therefore, a new strategy of selective blockade of pathological detrimental AT1R signaling is needed. In seeking for a new modulator of AT1R signaling, we and others previously identified the AT1R-associated protein (ATRAP/Agtrap), a molecule specifically interacting with the carboxyl- terminal domain of AT1R [2–5]. ATRAP selectively suppresses Ang II–mediated pathological hyper-activation of AT1R signaling, without any effect on baseline cardiovascular function including blood pressure (Fig. 1).

### Identification of ATRAP

The G protein-coupled receptors (GPCRs) interact with different classes of intracellular proteins, including heterotrimeric G proteins, kinases, and arrestins [6–8]. Although the intracellular third loop of a number of GPCRs plays an important role as a structural determinant of coupling of the receptor to heterotrimeric G proteins, accumulated experimental results also highlighted the functional importance of the carboxyl-terminal cytoplasmic domain in receptor signaling and internalization [9, 10]. Employing yeast two-hybrid screening of a mouse kidney cDNA library, with the carboxyl-terminal cytoplasmic domain of the mouse AT1R as a bait, a novel protein, with an open reading frame of 483 base pairs in its cDNA and with a predicted molecular mass of 18 kDa, was isolated and named ATRAP [2]. The ATRAP did not interact with the carboxyl-terminal cytoplasmic domains of the AT2R and those of several Gq-coupled receptors such as m3 muscarinic, bradykinin B2, and endothelin B receptors, nor did it associate with the Gq-coupled β2-adrenergic receptor. Thus, ATRAP is likely to be an AT1R-specific binding molecule to date. The human ATRAP cDNA was also cloned and the deduced polypeptide product of the cDNA was 22 kDa in size [11]. The human ATRAP cDNA and amino acid sequences were 85 and 77% identical to those of the mouse ATRAP gene, respectively.

### Predicted domain structure of ATRAP

Characterization using cultured cells revealed ATRAP as a transmembrane protein localized in intracellular trafficking vesicles and plasma membrane [2, 12]. With respect to the domain structure, ATRAP is predicted in silico to contain three hydrophobic domains at the amino-terminal end of the protein, encompassing the amino acid residues 14–36, 55–77, and 88–108 and a hydrophilic cytoplasmic carboxyl-terminal tail from residues 109–161. The first transmembrane domain consists of a mixture of apolar and polar amino acid residues; the second and third transmembrane domains are composed mainly of hydrophobic residues with some polar amino acid residues.

### Promoting effects of ATRAP on AT1R internalization

The results of the analysis of intracellular distribution of ATRAP showed a particulate distribution; electron microscopy reveals the presence of ATRAP in prominent perinuclear vesicular membranes; and co-localization analysis by immunofluorescence shows that ATRAP co-localizes in an intracellular vesicular compartment corresponding to endoplasmic reticulum, Golgi, and endocytic vesicles [12].

With respect to the interaction of ATRAP with AT1R and effects of ATRAP on AT1R internalization in cells, the results of immunoprecipitation assay, Bioluminescence resonance energy transfer (BRET) analysis, and immunofluorescence staining in cultured cells including cardiovascular cells indicated that ATRAP is able to interact with AT1R even without Ang II stimulation and that Ang II stimulation significantly facilitated the interaction of these proteins [4]. The results of real-time trafficking analysis of ATRAP vesicles also showed a constitutive translocation of ATRAP from intracellular vesicle compartments to the periphery of the cell, which was not affected by the treatment with Ang II [12].

Taken together, these results suggest that ATRAP is actually able to bind to the AT1R under baseline conditions but that ATRAP interacts mainly with the AT1R that is internalized from the cell surface into the endocytic vesicles on Ang II stimulation to keep the receptor internalized even after the removal of Ang II. Furthermore, the quantitative analysis of immunofluorescence staining indicated that almost all of the internalized AT1R were associated with ATRAP, indicating that a major function of ATRAP in cultured cells including cardiovascular cells is to promote the constitutive internalization of AT1R [4, 13, 14]. Furthermore, a transgenic model increase in renal ATRAP expression beyond the baseline in vivo was accompanied by a constitutive reduction of renal plasma membrane AT1R expression and by the promotion of renal AT1R internalization in response to Ang II [15]. In contrast, a genetic deficient model of ATRAP caused an enhanced surface expression of AT1R in the kidney [16].

### Putative functional role of ATRAP in cardiovascular cells

Initially, this protein has been found to modulate AT1R function in transformed African green monkey kidney fibroblast (COS-7) cells and human embryonic kidney (HEK) 293 cells [2, 12]. Overexpression of ATRAP in COS-7 cells caused a marked inhibition of AT1R-mediated activation of phospholipase C, and functional analysis of the effects of ATRAP on Ang II–induced AT1R signaling in HEK293 cells reveals a moderate decrease in the generation of inositol lipids, a marked decrease in Ang II-stimulated transcriptional activity of the c-fos promoter luciferase reporter gene, and a decrease in cell proliferation.

In cardiomyocytes, overexpression of ATRAP by adenoviral gene transfer significantly decreases the number of AT1R on the surface of cardiomyocytes, and it also decreases the degree of p38 mitogen–activated protein kinase (MAPK) phosphorylation, the activity of the c-fos promoter, and protein synthesis upon Ang II stimulation in cardiomyocytes. In addition, in vascular smooth muscle cells (VSMC) and in distal convoluted tubule cells (mDCT), overexpression of ATRAP inhibited Ang II–mediated increases in Transforming Growth Factor (TGF)-β mRNA expression and TGF-β production into the medium [4, 17]. On the other hand, ATRAP knockdown by small-interference RNA in VSMC activated Ang II-induced c-fos gene expression, which was effectively inhibited by valsartan, an AT1R-specific antagonist [4].

The nuclear factor of activated T cells (NFAT) transcription factor, which is dephosphorylated by the phosphatase calcineurin activated by the calcium signaling regulator and cyclophilin-binding protein, calcium-modulating cyclophilin ligand (CAML), has received broader interest in relation to various signaling events, in addition to regulating T cell receptor signaling [18]. It is expressed in cardiomyocytes, endothelial, and VSMC and is implicated in Ang II signaling through the AT1R [19]. Several findings have shown that the calcineurin/NFAT-signaling pathway induced by Ang II regulates cell growth and cardiovascular hypertrophy, contributing to pathological cardiovascular remodeling [20]. The CAML has been shown as an ATRAP partner, and the N-terminal hydrophilic domain of CAML (the amino acid residues 1–189) mediates a specific interaction between ATRAP and CAML. The amino acid residues 40–82 of ATRAP contribute to this interaction. Functionally, overexpression of ATRAP decreased Ang II–mediated and CAML–induced activation of calcineurin-NFAT pathway and inhibited cardiomyocyte hypertrophic response and VSMC senescence process [21, 22]. These results indicate that ATRAP significantly promotes the constitutive internalization of the AT1R and further attenuates certain Ang II–mediated pathological responses in cardiovascular and renal cells.

### Putative functional role of ATRAP in cardiovascular tissues

To examine the ATRAP-mediated effect on tissue AT1R internalization and AT1R signaling by a different strategy in vivo, several kinds of ATRAP transgenic mice were produced and analyzed to date. Cardiac-specific ATRAP transgenic mice were produced to examine a possible cardiac protective effect of ATRAP [23]. These ATRAP transgenic mice at baseline displayed no evident anatomical abnormality or alteration in physiological parameters, such as blood pressure and renal function. However, in cardiac-specific ATRAP transgenic mice, the development of cardiac hypertrophy, activation of p38 MAPK, and expression of hypertrophy-related genes in response to chronic Ang II infusion were suppressed, in spite of there being no significant difference in blood pressure between the transgenic mice and wildtype mice. These results demonstrate that cardiomyocyte-specific overexpression of ATRAP in vivo protected from the cardiac hypertrophy provoked by chronic Ang II infusion [23].

Transgenic model with a pattern of aortic vascular-dominant overexpression of ATRAP were also produced [24]. Ang II or vehicle was continuously infused into aortic vascular-dominant ATRAP transgenic mice and wild-type mice via an osmotic minipump for 14 days. Although blood pressure of Ang II-infused aortic vascular-dominant ATRAP transgenic mice was comparable to that of Ang II-infused wild-type mice, the Ang II-mediated development of aortic vascular injury was significantly suppressed in the aortic vascular-dominant ATRAP transgenic mice compared to wild type mice. In addition, the Ang II-mediated reactive oxygen species (ROS) generation was significantly suppressed in the aortic vascular-dominant ATRAP transgenic mice, with a concomitant inhibition of activation of aortic vascular p38 MAPK by Ang II. These results indicate that activation of aortic vascular ATRAP efficiently inhibits the ROS-p38MAPK pathway and pathological aortic hypertrophy provoked by Ang II-mediated hypertension [24].

### Putative functional role of ATRAP in kidney

ATRAP is broadly expressed in many tissues as is AT1R in vivo. Endogenous ATRAP protein is most abundantly expressed in the kidney, where it is highly expressed in tubular epithelial cells in proximal and distal tubules but only faintly expressed in glomeruli [17, 25]. To examine the functional role of ATRAP in the kidney, systemic ATRAP knockout mice were produced using a gene-targeting method [26, 27]. Systemic ATRAP knockout mice display no evident alteration in blood pressure and renal morphology/function at baseline. However, systemic ATRAP knockout mice exhibit exacerbation of angiotensin-dependent hypertension, concomitant with an increase in sodium retention [27]. Systemic ATRAP knockout mice also exhibit exacerbation of target organ damage such as cardiac hypertrophy and albuminuria, in response to Ang II. In systemic ATRAP knockout mice, renal expression of the sodium-proton antiporter 3 (NHE3), a major sodium transporter in the proximal tubules, was comparable to that of wild-type mice. However, Ang II-induced upregulation of epithelial sodium channel α-subunit (ENaCα), a major sodium transporter in distal tubules, was significantly enhanced in systemic ATRAP knockout mice compared with wild-type mice. There were no differences in blood pressure response and renal ENaC expression by aldosterone between systemic ATRAP knockout mice and wild-type mice [27].

Furthermore, we examined the functional role of ATRAP in suppressing hypertension in a mouse remnant kidney chronic kidney disease (CKD) model [16]. Systemic ATRAP knockout mice that underwent 5/6 nephrectomy showed hypertension with increased plasma volume. In systemic ATRAP knockout mice compared with wild-type mice after 5/6 nephrectomy, renal expression of the ENaCα and tumor necrosis factor (TNF)-α was significantly enhanced, concomitant with increased plasma membrane AT1R in the kidneys. In addition, TNF-α blockade with etanercept attenuates hypertension and renal expression of the epithelial sodium channel ENaCα in the remnant kidney model of systemic ATRAP deficient mice [16].

For gain-of-function in vivo strategy, ATRAP transgenic mice dominantly overexpressing ATRAP in renal tubules (renal ATRAP transgenic mice) were produced [28]. The renal ATRAP transgenic mice exhibited no significant change in blood pressure at baseline on normal salt diet. However, in contrast to systemic ATRAP knockout mice, renal ATRAP transgenic mice exhibit suppression of Ang II-induced hypertension, concomitant with a decrease in sodium retention compared with wild-type mice [28]. In addition, in the renal ATRAP transgenic mice compared with wild-type mice, the renal Na + -Cl − cotransporter (NCC) activation and ENaCα induction by Ang II infusion were inhibited [28]. The renal ATRAP transgenic mice also exhibit a suppression of blood pressure elevation and renal sodium reabsorption in response to high salt loading [29]. Functional transport activity of the amiloride-sensitive ENaC was significantly decreased under saline volume–expanded conditions in the renal ATRAP transgenic mice compared with wild-type mice [29].

Recently, we have reported the in vivo functional role of renal proximal tubule ATRAP in angiotensin-dependent hypertension [30]. Proximal tubule-specific ATRAP knockout mice were generated using the Cre/loxP system with *Pepck*-Cre. There were no significant differences in pressor response to angiotensin II infusion between proximal tubule-specific ATRAP knockout mice and wild-type mice. In addition, angiotensin II-mediated cardiac hypertrophy was identical between proximal tubule-specific ATRAP knockout mice and wild-type mice [27]. Collectively, the inhibitory effect of renal ATRAP on Ang II/CKD/salt hypertension appears to act mainly through a distal tubule ATRAP-mediated mechanism.

**Fig. 2:**
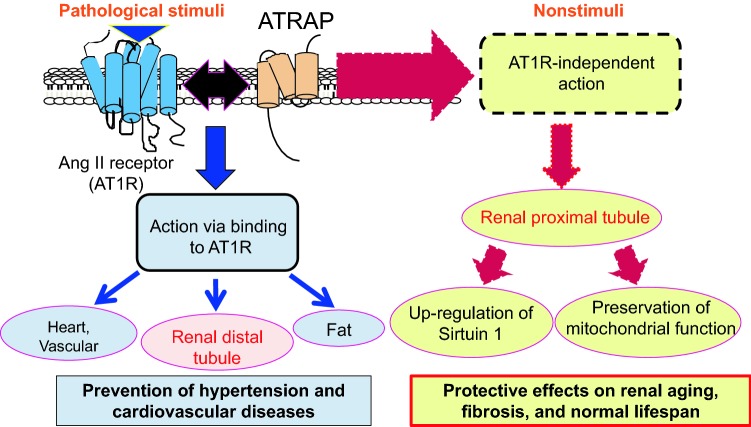
Angiotensin type 1 receptor-binding action and angiotensin receptor-independent action of ATRAP

Our hypothesis is that renal tubular ATRAP function may be different between distal tubules and proximal tubules (Fig. 2). Distal tubular ATRAP may have functionally selective inhibition of pathological detrimental AT1R signaling. On the other hand, proximal tubular ATRAP may have other function independent of AT1R signaling. We examined the in vivo functional role of ATRAP in the long-term process of aging using systemic ATRAP knockout mice [31]. Compared with wild-type mice, systemic ATRAP knockout mice show more advanced age-associated mitochondrial abnormalities and subsequently increased reactive oxygen species production in proximal tubules of the kidney, as well as exacerbated age-associated tubulointerstitial fibrosis. In addition, the lifespans of systemic ATRAP knockout mice is 18.4% shorter (median lifespan: 100.4 *vs.* 123.1 week) compared with wild-type mice. As a key mechanism, age-related pathological changes in the kidney of ATRAP-knockout mice correlated with decreased expression of the pro-survival gene *Sirtuin1* in renal proximal tubules [31]. On the other hand, chronic angiotensin II infusion did not affect renal sirtuin1 expression in wild-type mice. These results indicate that proximal tubular ATRAP plays an important role in inhibiting kidney aging possibly through sirtuin1-mediated mechanism independent of blocking AT1R signaling, and further protecting the normal lifespan.

### ATRAP expression in human

ATRAP is widely expressed in many tissues in human, as is AT1R [24, 25]. In the normal human kidney, both ATRAP mRNA and protein are abundantly distributed along the renal tubules from Bowman’s capsule to the medullary collecting ducts [16, 24]. In renal biopsy specimens from 22 patients with IgA nephropathy, a significant positive correlation between ATRAP and AT1R gene expression was observed [16]. In addition, there was a positive relationship between tubulointerstitial ATRAP expression and the estimated glomerular filtration rate in patients with IgA nephropathy [16]. ATRAP is also abundantly expressed in human adipose tissue [25]. In visceral adipose tissues from 36 patients who underwent abdominal surgery, ATRAP mRNA expression was significantly decreased in the adipose tissue from hypertensive patients compared with normotensive patients [25]. Similar trends of decrease in adipose ATRAP mRNA expression were observed in patients with obesity and diabetes. On the other hand, the adipose AT1R mRNA levels in patients with these metabolic disorders were the same as those in patients without respective metabolic disorders [25]. Furthermore, ATRAP is abundantly expressed in human leukocytes, predominantly in monocytes and granulocytes [31]. In 86 outpatients with non-communicable diseases, leukocyte ATRAP mRNA expression positively correlated with inflammatory parameters, such as the granulocyte and monocyte count, serum C-reactive protein and proinflammatory cytokine levels [31]. leukocyte ATRAP may be an emerging marker capable of reflecting the systemic and leukocyte inflammatory profile in the pathophysiology of non-communicable diseases.

## Conclusions and perspectives

Hyper-activation of tissue RAS through AT1R plays a pivotal role in the pathogenesis of hypertension and associated end-organ injury. On the other hand, physiological AT1R signaling is essential for maintaining organ homeostasis. ATRAP seems to be an endogenous inhibitor so as to suppress just the hyper-activation of AT1R signaling along with preserving the physiological activation of AT1R signaling. In addition, ATRAP may have an AT1R-independent function of renal proximal tubule to protect aging and fibrosis. We are going to seek for “Factor X” as a new partner of ATRAP, to elucidate the mechanism relevant to AT1R-independent function of ATRAP in renal proximal tubule. ATRAP is a possible target to modulate hypertension and cardiorenal/vascular diseases.
